# Cell surface antigens in renal tumour cells: detection by immunoluminescence and enzymatic analysis

**DOI:** 10.1054/bjoc.2001.2004

**Published:** 2001-09

**Authors:** F Laube, B Göhring, H Sann, I Willhardt

**Affiliations:** Institute of Physiological Chemistry, Hollystr. 1; Institute of Medical Immunology, Medical Faculty, Martin-Luther-University Halle-Wittenberg, Str. der OdF 6, Halle, D-06097, Germany

**Keywords:** renal cell carcinoma, CD44(var), ICAM-1, urokinase-type plasminogen activator, aminopeptidase N, immunoluminescence

## Abstract

Two renal cell carcinoma cell lines (49RC 43STR and 75RC 2STR) were characterized by detection of the cell surface proteins: CD44(var), intercellular adhesion molecule-1 (ICAM-1), urokinase-type plasminogen activator (uPA) and its receptor and aminopeptidase N (APN). To detect their localization the immunoluminescent technique was used. In addition, the enzyme activity of uPA and APN was investigated in cell suspensions as well as in monolayers. The latter procedure was more advantageous since the additional use of HPLC permits a single registration of the fluorescent hydrolysis-product AMC (7-amino-4-methylcoumarin) without interference by cellular autofluorescence or non-reacted fluorescent substrate. Unlike 75RC 2STR, the cell line 49RC 43STR expressed high levels of uPA and APN. Contrary to that the cell line 75RC 2STR expressed high levels of ICAM-1 and CD44(v6), whereas 49RC 43STR showed a low level of ICAM-1 and no distinct light signal with anti-CD44(v6). The uPA activity was measured directly as well as indirectly (via plasmin) with the substrate Z-Gly-Gly-Arg-AMC. Both activator and plasmin activity were inhibited by D-Val-Phe-Lys-CMK and phenylmethylsulfonyl fluoride. The anti-catalytic antibody to uPA and that to uPA receptor were found to be inhibiting the uPA activity in a concentration-dependent manner. APN activity was assayed using alanine-p-nitroanilide. Peptidase activity was effectively inhibited by 1,10-phenanthroline and partly inhibited by ethylenediamine-tetraacetic acid. © 2001 Cancer Research Campaignhttp://www.bjcancer.com


*British Journal of Cancer* (2001) **85**(6), 924–929
© 2001 Cancer Research Campaign
doi: 10.1054/ bjoc.2001.2004, available online at http://www.idealibrary.com on IDEAL

http://www.bjcancer.com

### Cell surface antigens in renal tumour cells: detection by immunoluminescence and enzymatic analysis

**F Laube[1], B Göhring[2], H Sann[1] and I Willhardt[1]**

[1]Institute of Physiological Chemistry, Hollystr. 1; [2]Institute of Medical Immunology, Str. der OdF 6, Martin-Luther-University Halle-Wittenberg, Medical Faculty, D-06097 Halle, Germany

**Summary** Two renal cell carcinoma cell lines (49RC 43STR and 75RC 2STR) were characterized by detection of the cell surface proteins: CD44(var), intercellular adhesion molecule-1 (ICAM-1), urokinase-type plasminogen activator (uPA) and its receptor and aminopeptidase N (APN). To detect their localization the immunoluminescent technique was used. In addition, the enzyme activity of uPA and APN was investigated in cell suspensions as well as in monolayers. The latter procedure was more advantageous since the additional use of HPLC permits a single registration of the fluorescent hydrolysis-product AMC (7-amino-4-methylcoumarin) without interference by cellular autofluorescence or non-reacted fluorescent substrate. Unlike 75RC 2STR, the cell line 49RC 43STR expressed high levels of uPA and APN. Contrary to that the cell line 75RC 2STR expressed high levels of ICAM-1 and CD44(v6), whereas 49RC 43STR showed a low level of ICAM-1 and no distinct light signal with anti-CD44(v6). The uPA activity was measured directly as well as indirectly (via plasmin) with the substrate Z-Gly-Gly-Arg-AMC. Both activator and plasmin activity were inhibited by D-Val-Phe-Lys-CMK and phenylmethylsulfonyl fluoride. The anti-catalytic antibody to uPA and that to uPA receptor were found to be inhibiting the uPA activity in a concentration-dependent manner. APN activity was assayed using alanine-p-nitroanilide. Peptidase activity was effectively inhibited by 1,10-phenanthroline and partly inhibited by ethylenediamine-tetraacetic acid. © 2001 Cancer Research Campaign http://www.bjcancer.com

**Keywords**: renal cell carcinoma; CD44(var); ICAM-1; urokinase-type plasminogen activator; aminopeptidase N; immunoluminescence

Specific CD44 isoforms, intercellular adhesion molecule-1, urokinase-type plasminogen activator and its receptor (uPA/uPAR) and aminopeptidase N (APN/CD13) are functionally important cell surface proteins involved in invasive and metastatic behaviour of tumour cells. At least 2 categories of proteins are responsible for invasion and metastasis: adhesion molecules and proteolytic enzymes. Adhesion proteins, as CD44(var) and ICAM-1 mediate cell–cell and cell–matrix interactions and proteases activate proenzymes or initiate whole proteolytic cascades resulting in the hydrolysis of extracellular matrix (ECM) and protein components of basement membranes. Among the tumour-associated proteinases and peptidases uPA and APN play a crucial role (Saiki et al, 1993; Riemann et al, 1995; Swiercz et al, 1998). Besides the primary biological function of uPA to catalyse the activation of plasminogen to plasmin this serine protease has a multifunctional potential. Associated with its receptor and the endogenous plasminogen activator inhibitor-type-1 (PAI-1) the following biological effects are described: (1) growth factor-like function, (2) mediation of cell adhesion and (3) signal-transduction function (Andreasen et al, 1997; Nguyen et al, 1998).

Compared to proteolytic enzymes which are secreted (i.e. released) in active or inactive form from the tumour cell into the ECM, this study was focused on membrane-bound proteases since they should be more effective with respect to invasion (Evans, 1991; Meissauer et al, 1992). It is generally suggested that proteases bound on the cell surface offer a more versatile and efficient attack on ECM in contrast to proteases that are released from the cell.

*Received 3 January 2001*
*Accepted 9 May 2001*

*Correspondence to:* F Laube

The aim of the present study was to analyse renal cell carcinoma (RCC) cells regarding the cell surface antigens mentioned above avoiding disadvantages of other methods (e.g. immunofluorescence: cellular autofluorescence).

Unlike CD44 variant sequences, ICAM-1 and uPAR – exclusively detected by immunoluminescence – uPA and APN were additionally characterized by enzyme activity assays. In contrast to tests of cell extracts or tissue homogenates by ELISA that can not differentiate between intracellular and extracellular antigen localization the immunological technique described here for intact cells enables one to detect the existence and extent of proteins on the cell surface (Laube, 1999a). Additionally, activity assays of vital tumour cells result in values for the specific proteolytic capacity that can be attributed to the tumour cell itself. This enzyme activity detected in cell suspensions or monolayers is not interfered by intracellular tumour enzymes or enzymes associated with or secreted from surrounding non-tumour cells as it is the case in tissue homogenates however.

### MATERIALS AND METHODS

### Cell lines

The human RCC cell lines 49RC 43STR and 75RC 2STR were originally provided by G Mickisch (Rotterdam, The Netherlands). Cells were cultured in RPMI 1640 medium containing $NaHCO_3$ (Biochrom, Berlin, Germany) and supplemented with L-glutamine, penicillin, streptomycin (Sigma, Deisenhofen, Germany) and 10% fetal calf serum (FCS) (CCPro, Neustadt/W, Germany). Cultures were maintained at 37˚C in a 5% $CO_2$ atmosphere. In order to obtain cell suspensions for the labelling of cell surface proteins standard culture flasks were used. To obtain cell monolayers, Petri dishes (diameter 35 mm, Greiner, Frickenhausen, Germany) were used for evaluation of the enzyme activity by HPLC.

### Antibodies

All monoclonal antibodies (mAbs) were of the IgG 1 type. The anti-CD44 mAbs were directed against the variable portion of the molecule: anti-CD44(v5), (VFF-8); anti-CD44(v6), (VFF-18); anti-CD44(v7), (VFF-9); polyclonal Ab: goat-anti-CD44(v3-v10) (Bender Med Systems, Vienna, Austria); anti-uPA (IM13L), anti-uPA(IM14L), anti-uPAR (GR35), anti-CD54 (15.2) (Calbiochem-Novabiochem, Bad Soden, Germany); anti-CD54 (HA58), anti-CD13 (WM15) (Pharmingen, Hamburg, Germany); anti-CD13 (SJ1D1) (Coulter-Immunotech, Marseille, France); conjugates: anti-mouse-IgG (Fab)-HRP and anti-sheep/goat-IgG (Fab)-HRP (Boehringer Mannheim, Germany). Normal mouse-IgG 1 (Sigma, Deisenhofen, Germany) and normal goat-IgG (Boehringer Ingelheim, Germany) were used for blocking and negative controls.

### Reagents

Luminol (3-aminophthalhydrazide), phenylmethylsulfonyl fluoride (PMSF) (Boehringer Mannheim, Germany); 6-hydroxybenzothiazole, plasminogen (from human plasma, P-5661), 1,10-phenanthroline, phosphatidylinositol-specific phospholipase C (PI-PLC) (Sigma, Deisenhofen, Germany); PI-PLC (recombinant, GPI-02) (Oxford GlycoSystems, UK); Z-Gly-Gly-Arg-AMC (urokinase substrate III), D-Val-Phe-Lys-CMK (chloro-methylketone) (Calbiochem-Novabiochem, Bad Soden, Germany); H-Ala-pNA (Bachem, Heidelberg, Germany).

### Cell labelling

Cells were grown to near confluence, harvested with EDTA/trypsin and washed with 0.01 M phosphate-buffered saline (PBS) pH 7.4. To reconstitute cell surface proteins, cells were resuspended and maintained in RPMI medium. Cells were washed thoroughly and incubated in PBS with normal goat-IgG (for the detection with specific mAbs) or normal mouse-IgG 1 (for the detection with the polyclonal goat-Ab) to block IgG and Fc receptors. Cells were washed and incubated with appropriate dilutions of the various Abs (Ab concentration generally used: 10 μg $ml^{-1}$; 60 min, 4˚C, with shaking) followed by washing the cells twice. Then, cells were incubated with the corresponding conjugate (45 min, 4˚C, with shaking) and after washing 3 times the cells were subjected to the luminescent test.

### Luminescence

Aliquots of cell suspension (100 μl, $5 \times 10^5$–$1 \times 10^6$ cells) were mixed with 200 μl substrate solution (PBS, pH 7.5; luminol/$H_2O_2$/6-hydroxybenzothiazole) (Kricka and Thorpe, 1990) and the light emission was measured as intensity (relative light units: RLU $s^{-1}$) 30 s after the initiation.

### Enzyme activities

### *uPA-activity assay*

(1) Endogenous membrane-bound uPA activity was assayed as described (Zimmerman et al, 1978) with modifications: the reaction was started by adding 100 μl of a cell suspension ($2 \times 10^6$ cells) to 1 ml substrate solution (Z-Gly-Gly-Arg-AMC, final concentration 0.15 mM in PBS) with or without inhibitor (D-Val-Phe-Lys-CMK, PMSF). Excitation and emission wavelengths were set at 380 nm and 450 nm, respectively. The fluorescence of AMC released was monitored continuously over a period of 5 min. Values for the maximal velocity (ΔRFU $min^{-1}$) were used for calculations. (2) An indirect assay for uPA activity was performed by release of plasmin from plasminogen (PG) after preincubation of cells with PG (final concentration 0.5 U $ml^{-1}$; 10 min, 37˚C) using the same procedure as described under (1).

### *APN-activity assay*

Tests were performed with intact cells freshly harvested and washed thoroughly. After 45 min preincubation of cells ($2 \times 10^6$/0.5 ml PBS, pH 7.4) in the presence or absence of different effectors (inhibitors: 1,10-phenanthroline, ethylenediamine-tetraacetic-acid-$Na_2$-salt (EDTA): 25˚C, Abs: 4˚C) 0.5 ml substrate solution was added (final concentration of Ala-pNA: 1.5 mM in PBS). Cells were allowed to react 20–30 min at 37˚C with gentle shaking. The amount of p-nitroaniline formed was measured in the supernatant by reading the absorbance at 405 nm. Each test was run in duplicate.

### High-pressure liquid chromatography (HPLC)

Cell monolayers grown in Petri dishes were used for uPA activity and inhibition assays (seeding: $1 \times 10^5$ cells/Petri dish in 2 ml medium; cultivation: 24 h in RPMI and additionally 24 h in RPMI without FCS; cell monolayers were rinsed thoroughly with PBS and subjected to the test). Endogenous membrane-bound uPA activity was assessed using the substrate Z-Gly-Gly-Arg-AMC (final concentration: 0.15 mM in PBS, 37˚C, reaction interval: 0–60 min). For kinetic analysis aliquots of the reactive supernatant from cell monolayers were collected, quickly frozen at –20˚C, and subsequently separated on a HPLC column. Column: Nucleosil 120/5, C-18, Macherey & Nagel, Germany); solvent system: acetonitrile/$H_2O$ = 60/40; $\lambda_{Ex}$ = 340 nm, $\lambda_{Em}$ = 440 nm. Recording: 8450 Fluorescence HPLC-Monitor (Bischoff, Germany). The increase of AMC released by the reaction was calculated with a software program (Euro-Chrom 2000 – Integration package, Knauer, Germany) using the peak area.

### Instrumentation

Luminescent detection was performed with the luminometer Lumat LB 9501 (Berthold, Germany), the fluorescent measurement in cell suspensions with the spectrophotometer F-2000 (Hitachi, Japan), and the colorimetric assay using the Zeiss spectrophotometer Spekol UV-VIS 3.01.

Cell viability was assessed by exclusion of trypan blue.

### RESULTS

Two human RCC cell lines were investigated for the expression of different tumour-specific cell surface proteins. Results obtained by immunolabelling are shown in Figure 1A–C. Due to more binding sites the polyclonal Ab to CD44(v3-v10) gave the most intensive signal. Only the cell line 75RC 2STR exhibited a strong reaction with anti-CD44(v6) and none of the cell lines displayed a distinct reaction with anti-CD44(v5) or antiCD44(v7). These results were reproducible and independent from culture stage and cell number.

© 2001 Cancer Research Campaign

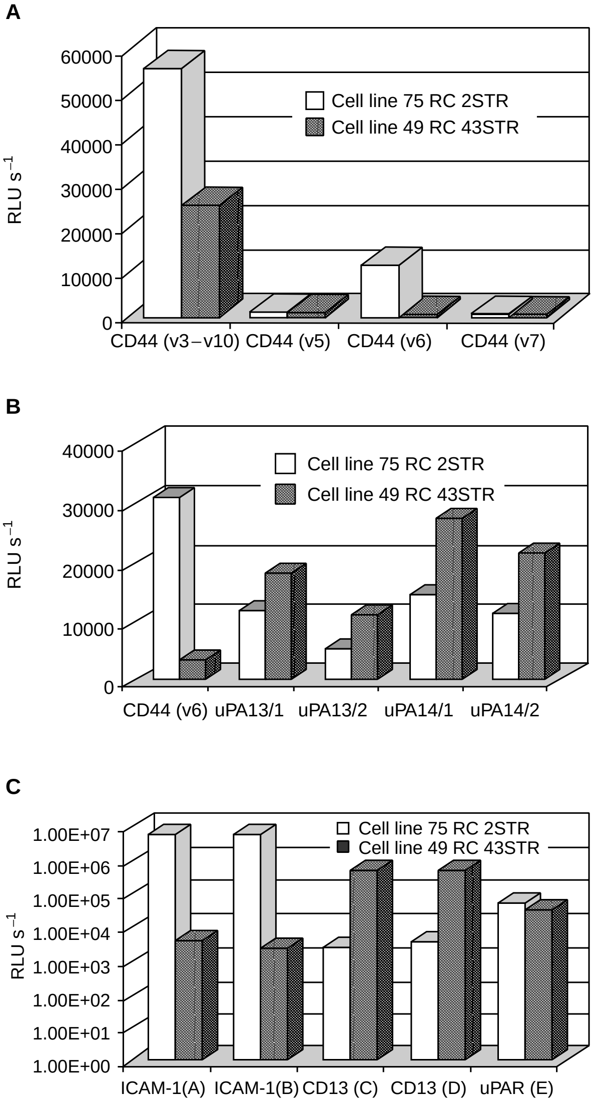

**Figure 1 (A–C)** Immunoluminescent detection of cell surface antigens in the renal cell carcinoma cell lines 75RC 2STR and 49RC 43STR. Values for the non-specific binding were subtracted. (**A**) Detection of CD44 variant sequences. (**B**) Detection of uPA in comparison with CD44(v6). The signal intensity for CD44(v6) of about 3000 RLU $s^{-1}$ (cell line 49RC 43 STR) is very low and not significant. 13: anti-uPA (IM13L) recognizes an epitope on the B-chain of uPA. 14: anti-uPA (IM14L) recognizes an epitope on the A-chain of uPA. 1: Ab concentration 10 μg $ml^{-1}$; 2: Ab concentration 2 μg $ml^{-1}$. (**C**) Detection of ICAM-1/CD54, APN/CD13 and uPAR/CD87. (A): anti-CD54 (15.2), (B): anti-CD54 (HA58), (C): anti-CD13 (SJ1D1), (D): anti-CD13 (WM15), (E): anti-uPAR (GR35)

Figure 1B shows the detection of uPA with 2 different mAbs compared to the signal intensity received for CD44(v6) in this series. In many labelling experiments anti-CD44(v6) was used as an internal control. The signal intensity for CD44(v6) of about 3000 RLU/s (49RC 43STR) is very low and not significant. The anti-uPA (IM13L) antibody recognizes an epitope on the B-chain which contains the catalytic active site of the molecule. The anti-uPA (IM14L) antibody recognizes an epitope in the amino terminal region of the A-chain. With identical Ab concentrations the anti-uPA (IM14L) antibody always exhibited a distinctly stronger signal compared to anti-uPA (IM13L) antibody. For both anti-uPA antibodies signal intensities were found to depend on the concentrations used (1 = 10 μg $ml^{-1}$ and 2 = 2 μg $ml^{-1}$, respectively).

Figure 1C demonstrates the immunological response obtained for ICAM-1, APN (CD13) and uPAR. Contrary to the level of uPAR that is nearly equal for both cell lines, the expression of ICAM-1 and APN is much different. While 49RC 43STR exhibit a high level of APN and a low level of ICAM-1, in the cell line 75RC 2STR for these 2 antigens an inverse proportion is observed.

In order to test partially the specific proteolytic capacity that can be attributed to the tumour cell itself, the activity of 2 typical proteases, uPA and APN, was investigated. Figure 2 shows a first approach to use intact cells for enzymatic assays. Using cell suspensions the uPA activity was measured directly with the fluorogenic urokinase substrate Z-Gly-Gly-Arg-AMC as well as indirectly via plasmin generated from human plasminogen. Details see under uPA activity assay, method (1) and (2), respectively. Because both uPA and plasmin are serine proteases with similar specificity the substrate Z-Gly-Gly-Arg-AMC was used for the detection of both proteases. For the cell line 49RC 43STR it was found that addition of plasminogen to a cell suspension results in an activity increase by 65% but the 75RC 2STR cells gave only an increase of about 10%. As Figure 2 clearly shows the activator activity as well as plasmin activity are inhibited by PMSF and the CMK-inhibitor (inhibition of uPA plus plasmin by PMSF is not shown). Because of the very low uPA expression by 75RC 2STR the activator activity and the plasmin activity of these cells are completely inhibited by D-Val-Phe-Lys-CMK.

Since the cell line 49RC 43STR showed the stronger immunological response and the higher activity level for uPA compared to 75RC 2STR, the former cells were used to test the uPA activity in cell monolayers grown in Petri dishes (Figure 3). Aside from the partial inhibition by PMSF and the CMK-inhibitor the uPA activity was markedly inhibited following the preincubation with specific Abs to uPA (B-chain) and uPAR. Both Abs reduce the uPA activity in a concentration-dependent manner. But the Ab to an epitope on the B-chain, bearing the active site, diminishes the activity to a higher degree than the Ab to the receptor. Using mouse non-immune IgG 1 as control the uPA activity was decreased negligible. Because of the GPI anchor-bound uPAR PI-PLC was used to release the uPA/uPAR complex from the cell. However, the uPA activity of PI-PLC-pretreated cells was decreased only 10–15%. Possible reasons for the hindered accessibility of the anchor-bound receptor will be discussed below.

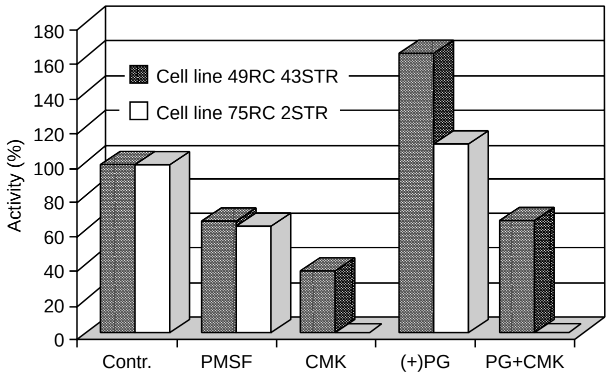

**Figure 2** uPA activity determined by method (1) and (2), respectively. Contr.: without inhibitor; PG: plasminogen; PG+CMK: inhibited enzyme activity assay following preincubation of cells with PG. CMK (D-Val-Phe-Lys-CMK) and PMSF were used in a final concentration of 0.1 mM. For further details see under Materials and methods

 © 2001 Cancer Research Campaign

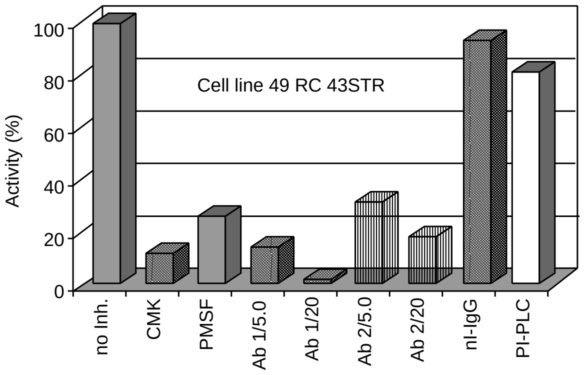

**Figure 3** uPA activity determined in cell monolayers by HPLC. Each value represents the AMC released after 40 min substrate reaction at 37˚C. The AMC peak areas obtained by HPLC were used for quantification. CMK (D-Val-Phe-Lys-CMK) and PMSF were used in a final concentration of 0.1 mM. Ab 1: anti-uPA (IM13L), Ab 2: anti-uPAR (GR35), Ab preincubation: 45 min, 4˚C, Ab concentration: 5.0: 5 μg ml$^{-1}$, 20: 20 μg ml$^{-1}$, nI-IgG: mouse non-immune IgG 1 (20 μg ml$^{-1}$). PI-PLC: phosphatidylinositol-specific phospholipase C (preincubation: 1 h, 37˚C, PBS pH 7.4)

On the other hand it could be shown by a brief treatment (30 s) of cells with glycine buffer, pH, 3, 0 (Cubellis et al, 1990; Mohanam et al, 1993; Hoyer-Hansen et al, 1997) that the uPA activity was decreased to 68.2% in 75RC 2STR and to 33% in 49RC 43STR cells due to the removal of uPA from the receptor (results not shown).

In spite of the lower level of APN based on the immunological result (Figure 1C) the cell line 75RC 2STR was used for the activity assay because of the more moderate enzyme activity (Figure 4). In the cell line 49RC 43STR APN is overexpressed and therefore the intense reactivity of a given cell number is not favourable to observe distinct inhibition effects. From the numerous inhibitors suitable for the metalloprotease APN ($Zn^{2+}$-dependent), 1,10-phenanthroline and EDTA were employed. Both inhibitors decrease the APN activity in a concentration-dependent fashion, but, despite lower concentrations used, 1,10-phenanthroline to a higher degree than EDTA. Further it was shown that both mAbs (up to a concentration of 20 μg ml$^{-1}$) used for the detection of APN only caused an inhibitory effect of about 15% (results not shown).

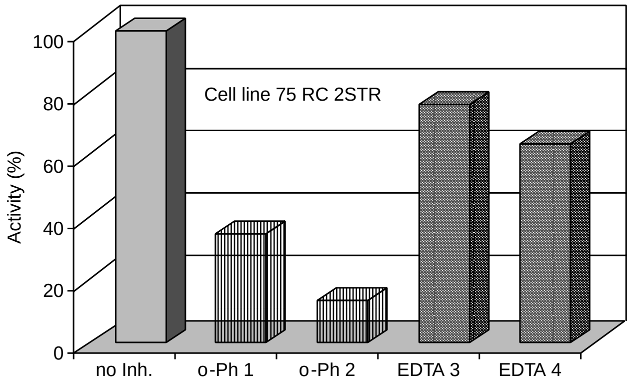

**Figure 4** APN activity assay. o-Ph: 1, 10-phenanthroline. The final concentrations of the inhibitors were: 1: 0.5 mM, 2: 1.5 mM, 3: 1.0 mM, 4: 2.0 mM

### DISCUSSION

This investigation was performed in order to characterize 2 RCC cell lines by immunological labelling as well as enzyme activity assays. For that purpose several cell surface antigens with close relations to cell–cell and cell–matrix adhesion and ECM breakdown were selected: CD44 isoforms, ICAM-1, uPA/uPAR and APN.

So far, numerous investigations of cell surface proteins were performed by immunofluorescent methods (e.g. flow cytometry) or immunohistochemistry. Previous experiments have shown that cell surface proteins can also be detected by immunoluminescence (Laube, 1996; Lischke et al, 1996; Laube and Göhring, 1997). This contribution describes the application of immunoluminescence for the detection of surface antigens mentioned above on intact cells. It is possible to use this advantageous technique as an alternative method to immunofluorescent labelling. The immunoluminescent technique is reproducible and very sensitive. After immunolabelling of cells using horseradish peroxidase (HRP) as marker enzyme for the first or second Ab this method enables both luminescent and fluorescent measurements. HRP substrates are available to produce luminescence (luminol) or fluorescence (3-(p-hydroxyphenyl) propionic acid and 10-acetyl-3,7-dihydroxyphenoxazine), (Laube, 1999a). Many applications of immunoassays associated with chemiluminescence are described but only few contributions have been published concerning the detection of cell surface proteins of viable cells. Indeed, immunoluminescence is based on a similar procedure as immunofluorescence, but the former has some advantages: there is no need for fluorochrome conjugates (fluorochrome-labelled Abs), light excitation is not required, that means photobleaching and cellular autofluorescence do not occur (non-transfected mammalian cells do not produce *bio*luminescence), simple control to check cells for endogenous peroxidase-like proteins that would interfere with the test, and easy measurement with a low-cost tube luminometer compared to an expensive flow cytometer.

This contribution has shown that all 5 membrane-bound antigens are expressed to a different degree in the 2 RCC cell lines. The activity assays, additionally performed to immunoluminescent labelling, enabled us to confirm the individual immunological signal intensity: a low immunological response of a given antigen (protease) conformed with a moderate to little enzyme activity. Unlike 75RC 2STR the cell line 49RC 43STR expressed high levels of APN and uPA. Contrary to that the cell line 75RC 2STR expressed high levels of ICAM-1 and CD44(v6), whereas 49RC 43STR showed a low level of ICAM-1 and no distinct light signal with anti-CD44(v6).

A comparison of different tumours demonstrates distinct variations in the CD44 isoform expression profile. Reasons for inconsistent results even within the RCC group are discussed in a critical manner (Hara et al, 1999). The authors found that the standard CD44 isoform (CD44s) is present in normal kidneys and in most RCCs but the incidence of predominant expression of CD44(v8-v10) in high-stage RCC is significantly higher than that in low-stage RCC. In paraffin-embedded tumour tissues the CD44(v6) variant sequence was detected in 2 out of 91 (Paradis et al, 1999) and in 6 out of 27 samples (Heider et al, 1996).

Since CD44 mainly mediates cell attachment to extracellular matrix components (especially to hyaluronat, HA) and to HA-bearing cells the importance of CD44 modulation by alternative splicing could be in a higher stage of progression rather than in the

© 2001 Cancer Research Campaign initiation of RCC. Finally, in a late stage of carcinogenesis an increased CD44 expression may allow tumour cells to attach more tightly to HA in the target tissue promoting tumour cell implantation (Gilcrease et al, 1996; Penno and Hart, 1998). If results of melanoma cells are to be included into this matter more complex conditions are revealed for possible mechanisms. The dominant features of highly aggressive melanoma cells compared to less aggressive melanomas and melanocytes were: (1) the higher level of CD44 epitope recognized by the mAb Hermes-1; (2) phosphorylation of the CD44 molecule; (3) a significantly higher rate of CD44 synthesis; (4) shedding of CD44 from and secretion of HA by the tumour cells (Goebeler et al, 1996).

Unlike CD44 ICAM-1 mediates heterotypical cell–cell interactions especially to cells bearing the lymphocyte function-related antigen-1 (LFA-1). ICAM-1 may facilitate the attachment of tumour cells to vascular endothelium following to invade the basement membrane. Another effect of tumour cells expressing ICAM-1 is the reduced interaction with lymphocytes by (1) decreased expression of HLA class I molecules (major histocompatibility complex), (2) secretion of immunsuppressive cytokines (e.g. interleukin 10) and (3) release of soluble ICAM-1 that competes for its receptor (LFA-1) on the lymphocyte to prevent the attachment via membrane-bound ICAM-1. These features enable one to evade the normal host immune response by the tumour cell. Cell surface expression and shedding of ICAM-1 by RCCs is described on stimulated cells (Hansen et al, 1994; Tanabe et al, 1997) and unstimulated cells (Koga et al, 1997).

uPA associated with tumour is described as a multifunctional factor (Andreasen et al, 1997; Reuning et al, 1998). The standard mechanism commonly accepted for receptor-bound uPA is the release of plasmin from plasminogen and the following activation of matrix metalloproteinases (MMPs) such as type IV pro-collagenase by plasmin. Subsequently, plasmin as well as activated MMPs may degrade ECM proteins facilitating invasion through tissue barriers (Ellis and Dano, 1998). Investigation of 18 RCC tissue samples revealed an uPA antigen content twice as much as compared with normal kidney or tumour-adjacent tissue and the uPA-like activity was triple that of normal control tissue (Kirchheimer et al, 1985). Using immunohistochemistry and ELISA techniques uPA/uPAR was analysed in 33 RCC tissue samples (Wagner et al, 1995). They found that uPA is expressed at relatively low levels in RCCs without significant difference to normal non-tumourous kidney tissue. The authors explain the similar distribution of uPA in tumourous and normal kidney tissue by the usually high endogenous level in healthy kidney that may disguise uPA changes in RCC. Without use of an uPA activity assay these authors were unable to differentiate between enzymatically active uPA and inactive pro-uPA in RCC. Secondly, a speculation was made to explain the difference between the low uPA mRNA level and the distinctly higher uPA protein level in RCC: uPA receptors on RCC cells are capable of binding uPA molecules secreted from surrounding non-tumour cells. On the other hand, a significantly higher expression of all components of the uPA activation system (uPA, uPAR and PAI-1) in kidney cancers compared with normal kidney tissue was found using the ELISA technique (Swiercz et al, 1998). Another investigation has shown the influence of different organs and their fibroblasts on the production of uPA by human RCC cells implanted in nude mice (Gohji et al, 1997). High levels of uPA were produced by the metastatic kidney tumours and lung metastases, whereas the subcutaneous tumours produced low levels. RCC cells co-cultured with mouse kidney or lung fibroblasts produced higher levels of uPA than tumour cells co-cultured with mouse skin fibroblasts. These findings suggest that the expression of uPA on tumour cells is influenced by surrounding non-tumour cells and their secreted factors.

On tumour cells uPA/uPAR is present at focal adhesion sites, preferentially at the invasive front and in contact to substratum (Andreasen et al, 1997), thus on adherent growing cells most of the uPA/uPAR complex is hidden. Additionally, human melanoma cells were shown to have an uPAR/uPA localization described as present in caveolae, flask-shaped microinvaginations of the plasma membrane (Stahl and Mueller, 1995). The negligible reactivity of the PI-PLC (Figure 3) may be caused by the hidden localization of the uPAR/uPA as well as the existence of a ‘screening’ glycocalyx (Koelsch et al, 1994). Extensive summaries of the role of APN in tissue invasion and its adhesive ability on various tumour cells were given (Nanus et al, 1997; Noren et al, 1997). The detection of APN antigen as well as APN activity in RCCs and RCC cells was described in detail (Saiki et al, 1993; Riemann et al, 1995; Göhring et al, 1998).

To summarize, additional to the proteolytic capacity of cells expressing uPA/uPAR and APN some findings clearly show an adhesive contribution of these cell surface proteins (Stahl and Mueller, 1997; Reuning et al, 1998). This multifunctional capacity of some cell surface proteins and their modulation suggests that different tumour cells could exhibit a similar adhesive and metastatic behaviour in spite of certain variations of the surface antigen composition. Extensive immunophenotyping of tumour cells including activity or functional tests could reveal whether a low-expressed or absent protein(ase) could be replaced by another equivalent functioning protein(ase).

Examples mentioned above clearly show that a lot of factors confer aggressive and metastatic behaviour to tumour cells. But crucial regulatory mechanisms responsible for growth and metastasis are still unclear. Investigations of more than one functional component of a tumour cell in combination with activity or functional tests should provide more insight into these complex processes (Laube, 1999b; Yu and Stamenkovic, 1999).

### REFERENCES

Andreasen PA, Kjoller L, Christensen L and Duffy MJ (1997) The urokinase-type plasminogen activator system in cancer metastasis: A Review. *Int J Cancer* **72**: 1–22

Cubellis MV, Wun TC and Blasi F (1990) Receptor-mediated internalization and degradation of urokinase is caused by its specific inhibitor PAI-1. *EMBO J* **9**: 1079–1085

Ellis V and Dano K (1998) u-Plasminogen activator. In: *Handbook of Proteolytic Enzymes*, Barrett AJ, Rawlings ND and Woessner JF (eds) pp 177–184. Academic Press: San Diego

Evans CW (1991) *The Metastatic Cell. Behaviour and Biochemistry*, pp 206–214. Chapman and Hall: London

Gilcrease MZ, Truong L and Brown RW (1996) Correlation of very late activation integrin and CD44 expression with extrarenal invasion and metastasis of renal cell carcinomas. *Human Pathol* **27**: 1355–1360

Goebeler M, Kaufmann D, Bröcker EB and Klein CE (1996) Migration of highly aggressive melanoma cells on hyaluronic acid is associated with functional changes, increased turnover and shedding of CD44 receptors. *J Cell Science* **109**: 1957–1964

Göhring B, Holzhausen HJ, Meye A, Heynemann H, Rebmann U, Langner J and Riemann D (1998) Endopeptidase 24.11/CD10 is down-regulated in renal cell cancer. *Int J Mol Med* **2**: 409–414

Gohji K, Nakajima M, Boyd D, Dinney CPN, Bucana CD, Kitazana S, Kamidono S and Fidler IJ (1997) Organ-site dependence for the production of urokinase-type plasminogen activator and metastasis by human renal cell carcinoma cells. *Am J Pathol* **151**: 1655–1661 927

 © 2001 Cancer Research Campaign

Hansen AB, Lillevang ST and Andersen CB (1994) Stimulation of intercellular adhesion molecule-1 (ICAM-1) antigen expression and shedding by interferon-γ and phorbol ester in human renal carcinoma cell cultures: relation to peripheral blood mononuclear cell adhesion. *Urol Res* **22**: 85–91

Hara I, Miyake H, Yamanaka K, Hara S, Arakawa S and Kamidono S (1999) Expression of CD44 adhesion molecules in nonpapillary renal cell carcinoma and normal kidneys. *Urol* **54**: 562–566

Heider KH, Ratschek M, Zatloukal K and Adolf GR (1996) Expression of CD44 isoforms in human renal cell carcinomas. *Virchows Arch* **428**: 267–273

Hoyer-Hansen G, Ploug M, Behrendt N, Ronne E and Dano K (1997) Cell-surface acceleration of urokinase-catalyzed receptor cleavage. *Eur J Biochem* **243**: 21–26

Kirchheimer JC, Pflüger H, Hienert G and Binder BR (1985) Increased urokinase activity to antigen ratio in human renal-cell carcinoma. *Int J Cancer* **35**: 737–741

Koelsch R, Gottwald S and Lasch J (1994) Release of GPI-anchored membrane aminopeptidase P by enzymes and detergents has some peculiarities. *Biochim Biophys Acta* **1190**: 170–172

Koga H, Naito S, Nakashima M, Hasegawa S, Watanabe T and Kumazawa J (1997) A flow cytometric analysis of the expression of adhesion molecules on human renal cell carcinoma cells with different metastatic potentials. *Eur Urol* **31**: 86–91

Kricka LJ and Thorpe GHG (1990) Bioluminescent and chemiluminescent detection of horseradish peroxidase labels in ligand binder assays. In *Luminescence Immunoassay and Molecular Applications*, Van Dyke K and Van Dyke R (eds) pp 77–98. CRC Press: Boca Raton

Laube F (1996) Immunoluminescence detection of cathepsin L/procathepsin L at the surface of human lung tumor cells. *Biol Chem* **377**: S 138

Laube F and Göhring B (1997) Detection of CD44 at the cell surface of human tumor cells by immunoluminescence. *Eur J Clin Chem Clin Biochem* **35**: A 112

Laube F (1999a) Cell surface expression of CD44: Detection on tumor cells by immunoluminescence compared to fluorescence analysis. In *Bioluminescence and Chemiluminescence: Perspectives for the 21st Century*, Roda A, Pazzagli M, Kricka LJ and Stanley PE (eds) pp 126–129. J. Wiley & Sons: Chichester

Laube F (1999b) Co-Localization of CD44 and urokinase-type plasminogen activator (uPA) on the surface of human melanoma cells. *Anticancer Res* **19**: 5709–5710

Lischke A, Pagany M, Kammer W and Friedrich K (1996) A chemiluminescence-based method for the detection and quantification of antigen-antibody interactions on the surface of eukaryotic cells. *Anal Biochem* **236**: 322–326

Meissauer A, Kramer MD, Schirrmacher V and Brunner G (1992) Generation of cell surface-bound plasmin by cell associated urokinase-type or secreted tissue-type plasminogen activator: A key event in melanoma invasiveness in vitro. *Exp Cell Res* **199**: 179–190

Mohanam S, Sawaya R, McCutcheon I, Ali-Osman F, Boyd D and Rao JS (1993) Modulation of in vitro invasion of human glioblastoma cells by urokinase-type plasminogen activator receptor antibody. *Cancer Res* **53**: 4143–4147

Nanus DM, Papandreou CN and Albino AP (1997) Expression of cell-surface peptidases in neoplastic cells. In *Cell-Surface Peptidases in Health and Disease*, Kenny AJ and Boustead CM (eds) pp 353–369. BIOS Scientific Publishers: Oxford

Nguyen DHD, Hussaini IM and Gonias SL (1998) Binding of urokinase-type plasminogen activator to its receptor in MCF-7 cells activates extracellular signal-regulated kinase 1 and 2 which is required for increased cellular motility. *J Biol Chem* **273**: 8502–8507

Noren O, Sjöström H and Olsen J (1997) Aminopeptidase N. In *Cell-Surface Peptidases in Health and Disease*, Kenny AJ and Boustead CM (eds) pp 175–191. BIOS Scientific Publishers: Oxford

Paradis V, Ferlicot S, Ghannam E, Zeimoura L, Blanchet P, Eschwege P, Jardin A, Benoit G and Bedossa P (1999) CD44 is an independent prognostic factor in conventional renal cell carcinomas. *J Urol* **161**: 1984–1987

Penno MB and Hart JC (1998) CD44, hyaluronan and lung cancer. In *Cell Adhesion Molecules and Matrix Proteins*, Mousa SA (ed) pp 175–190. Springer-Verlag: Berlin

Reuning U, Magdolen V, Wilhelm O, Fischer K, Lutz V, Graeff H and Schmitt M (1998) Multifunctional potential of the plasminogen activation system in tumor invasion and metastasis. *Int J Oncol* **13**: 893–906

Riemann D, Kehlen A and Langner J (1995) Stimulation of the expression and the enzyme activity of aminopeptidase N/CD13 and dipeptidylpeptidase IV/CD26 on human renal cell carcinoma cells and renal tubular epithelial cells by T cell-derived cytokines, such as IL-4 and IL-13. *Clin Exp Immunol* **100**: 277–283

Saiki I, Fujii H, Yoneda J, Abe F, Nakajima M, Tsuruo T and Azuma I (1993) Role of amino peptidase N (CD13) in tumor-cell invasion and extracellular matrix degradation. *Int J Cancer* **54**: 137–143

Stahl A and Mueller BM (1995) The urokinase-type plasminogen activator receptor, a GPI-linked protein, is localized in caveolae. *J Cell Biol* **129**: 335–344

Stahl A and Mueller BM (1997) Melanoma cell migration on vitronectin: regulation by components of the plasminogen activation system. *Int J Cancer* **71**: 116–122

Swiercz R, Wolfe JD, Zaher A and Jankun J (1998) Expression of the plasminogen activation system in kidney cancer correlates with its aggressive phenotype. *Clin Cancer Res* **4**: 869–877

Tanabe K, Campbell SC, Alexander JP, Steinbach F, Edinger MG, Tubbs RR, Novick AC and Klein EA (1997) Molecular regulation of intercellular adhesion molecule 1 (ICAM-1) expression in renal cell carcinoma. *Urol Res* **25**: 231–238

Wagner SN, Atkinson MJ, Thanner S, Wagner C, Schmitt M, Wilhelm O, Rotter M and Höfler H (1995) Modulation of urokinase and urokinase receptor gene expression in human renal cell carcinoma. *Am J Pathol* **147**: 183–192

Yu Q and Stamenkovic I (1999) Localization of matrix metalloproteinase 9 to the cell surface provides a mechanism for CD44-mediated tumor invasion. *Genes & Dev* **13**: 35–48

Zimmerman M, Quigley JP, Ashe B, Dorn C, Goldfarb R and Troll W (1978) Direct fluorescent assay of urokinase and plasminogen activators of normal and malignant cells: Kinetics and inhibitor profiles. *Proc Natl Acad Sci USA* **75**: 750–753

© 2001 Cancer Research Campaign 
